# Development and validation of a checklist for use with automatically generated radiotherapy plans

**DOI:** 10.1002/acm2.13694

**Published:** 2022-06-30

**Authors:** Kelly A. Nealon, Laurence E. Court, Raphael J. Douglas, Lifei Zhang, Eun Young Han

**Affiliations:** ^1^ University of Texas MD Anderson UTHealth Graduate School of Biomedical Sciences Houston Texas USA; ^2^ Department of Radiation Physics – Research The University of Texas MD Anderson Cancer Center Houston Texas USA; ^3^ Department of Radiation Physics – Patient Care The University of Texas MD Anderson Cancer Center Houston Texas USA

**Keywords:** automation, checklist, patient safety, quality assurance, risk assessment

## Abstract

**Purpose:**

To develop a checklist that improves the rate of error detection during the plan review of automatically generated radiotherapy plans.

**Methods:**

A custom checklist was developed using guidance from American Association of Physicists in Medicine task groups 275 and 315 and the results of a failure modes and effects analysis of the Radiation Planning Assistant (RPA), an automated contouring and treatment planning tool. The preliminary checklist contained 90 review items for each automatically generated plan. In the first study, eight physicists were recruited from our institution who were familiar with the RPA. Each physicist reviewed 10 artificial intelligence‐generated resident treatment plans from the RPA for safety and plan quality, five of which contained errors. Physicists performed plan checks, recorded errors, and rated each plan's clinical acceptability. Following a 2‐week break, physicists reviewed 10 additional plans with a similar distribution of errors using our customized checklist. Participants then provided feedback on the usability of the checklist and it was modified accordingly. In a second study, this process was repeated with 14 senior medical physics residents who were randomly assigned to checklist or no checklist for their reviews. Each reviewed 10 plans, five of which contained errors, and completed the corresponding survey.

**Results:**

In the first study, the checklist significantly improved the rate of error detection from 3.4 ± 1.1 to 4.4 ± 0.74 errors per participant without and with the checklist, respectively (*p *= 0.02). Error detection increased by 20% when the custom checklist was utilized. In the second study, 2.9 ± 0.84 and 3.5 ± 0.84 errors per participant were detected without and with the revised checklist, respectively (*p* = 0.08). Despite the lack of statistical significance for this cohort, error detection increased by 18% when the checklist was utilized.

**Conclusion:**

Our results indicate that the use of a customized checklist when reviewing automated treatment plans will result in improved patient safety.

## 1 INTRODUCTION

Radiotherapy is a complicated treatment technique that is used to treat approximately half of all cancer patients.^1^ Radiotherapy requires several components: a CT image of the patient's anatomy, manually or automatically created contours to identify targets and organs at risk, and a treatment plan generated using complex algorithms to model the patient dose. Each step is susceptible to error; as such, a thorough review of the final treatment plan must be performed to limit patient risk. This includes a physics plan review of many aspects of the treatment plan, including patient information, plan dosimetry, and treatment parameters.^2^, ^3^

According to a study by Ford et al., a physics pretreatment plan review is the step of the planning process that is most likely to detect errors before they impact patient treatment.^4^ Recommendations have been made regarding the content, frequency, and methods of plan reviews to maximize effectiveness.^5^ Checklists have been shown to improve the rate of error detection.^6^, ^7^, ^8^, ^9^, ^10^

While American Association of Physicists in Medicine (AAPM) task group 275 provides recommendations on how to perform a physics plan review, this report was written prior to the automation boom that is currently occurring in radiotherapy.^11^ New treatment planning tools automate aspects of the planning process, including contouring, planning, and quality assurance.^12^, ^13^, ^14^, ^15^, ^16^, ^17^, ^18^, ^19^, ^20^, ^21^ Automation can streamline the process, limiting the need for human interaction and decreasing the planning time.^22^, ^23^, ^24^ While this lack of human input could limit human error, it could also decrease the error detection rate because of the lack of human review. Because of the different workflows used in automated contouring and treatment planning tools, the effectiveness of manual checklists in the physics review process, specifically in automated plans, should be evaluated.

In this study, we developed a customized checklist to improve the rate of errors detected during the review of treatment plans that had been automatically generated by the Radiation Planning Assistant (RPA), an automated contouring and treatment planning tool that is currently under development.^15^ Planning errors were simulated, and the physics plan review was performed both without and with the custom checklist. Based on feedback from reviewers, the checklist was modified to optimize the effectiveness for use with automatically generated plans. Although it was tested with a specific automated process (the RPA), the study results will apply to the automated processes that are increasingly available in commercial treatment planning systems.

## 2 METHODS AND MATERIALS

### 2.1 Checklist development

**FIGURE 1:**
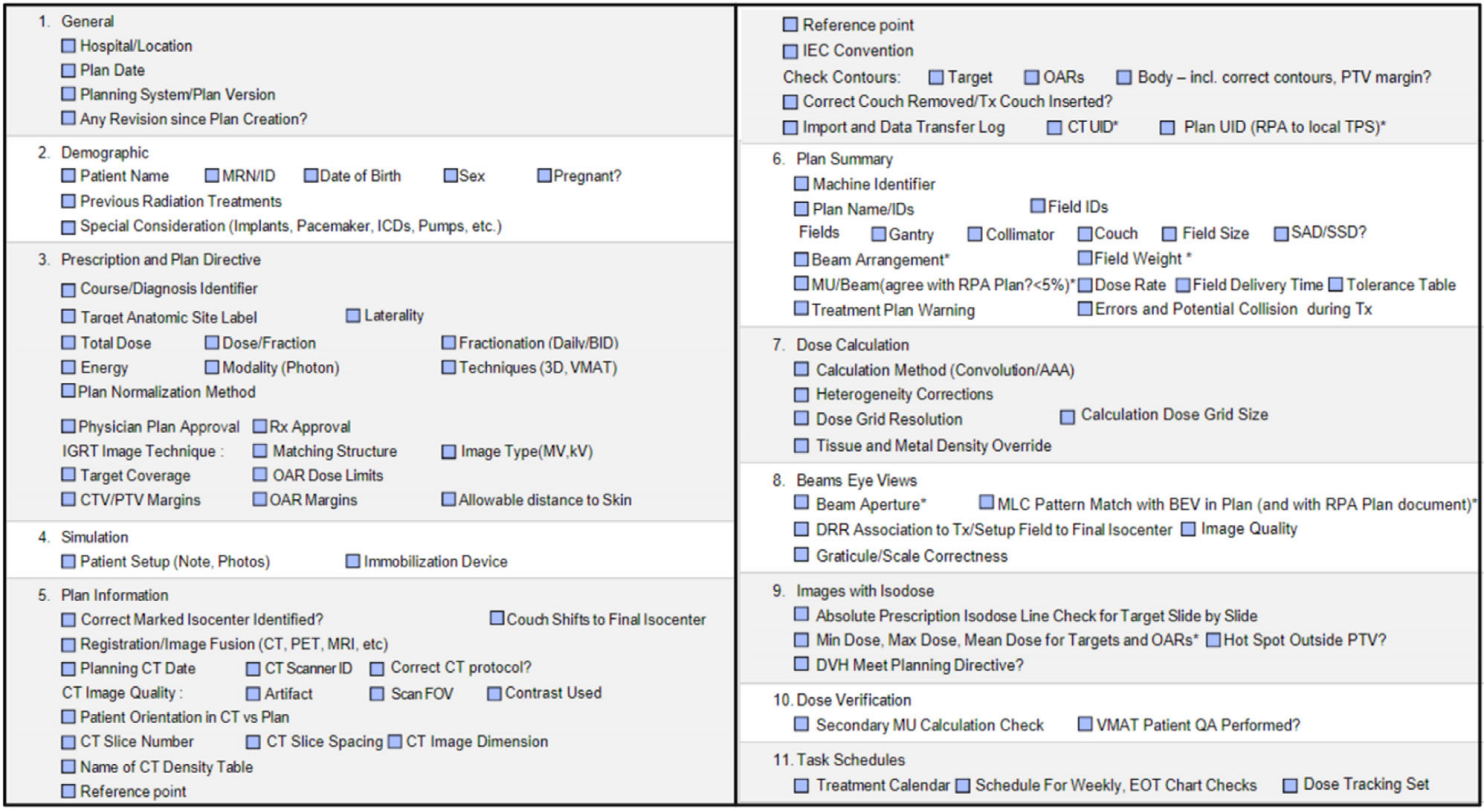
Checklist (version 1). Items for review were included based on recommendations from AAPM TG‐275 and TG‐315 and the results of a failure mode and effects analysis of the Radiation Planning Assistant (RPA). 90 total items were included to be reviewed

A customized plan review checklist was developed using guidance from AAPM task groups 275 and 315 (Medical Physics Practice Guideline 11.a).^3^, ^5^ Based on the results of a failure modes and effects analysis of the clinical integration of the RPA, the checklist was modified to address additional high‐risk points of error.^25^ This checklist directly addresses known, common, and critical errors which could occur in the RPA planning process. The preliminary checklist (Figure 1) contained 90 items to be checked for each RPA‐generated plan; these fell into the categories of general, demographic, prescription and plan directive, simulation, plan information, plan summary, dose calculation, beam's eye views, isodose images, dose verification, and task scheduling. This comprehensive checklist was reviewed by two physicists and several developers from the RPA team, to verify clarity before proceeding.

### 2.2 Study 1

To evaluate the effectiveness of our plan review checklist we assembled a group of eight physicists from MD Anderson with at least 2 years of clinical experience, including review of external beam radiotherapy treatment plans. These physicists were also provided with training on how to safely use the RPA as part of this study. The training included videos providing step‐by‐step instructions for how to generate treatment plans in the RPA, as well as how to review the final plan report. These videos discuss what errors could occur in the plan generation process, and how to detect them in the final plan and report. Users were also provided with all user documentation for the RPA planning system. Participants were instructed to review all training materials and to follow up if they had any questions.

We provided each physicist with 10 automatically generated treatment plans (four cervical cancer plans, three chest wall plans, and three head and neck plans) and imported them into RayStation, along with the corresponding RPA plan report as a PDF file. Details of the automatic algorithms used to generate the plans are described elsewhere.^16^, ^17^, ^26^ Of the 10 plans provided to each reviewer, five contained deliberate errors, all of which were identified as high risk in our failure modes and effects analysis study.^25^ These errors included incorrect treatment laterality, unidentified isocenter, incorrect coverage of the target, inappropriate dose normalization, and incorrect placement of the reference point and were introduced in the automated planning process.

The 8 physicists performed plan quality and safety checks according to their normal process for the 10 automatically generated treatment plans without the checklist and recorded any errors that they found. They also rated the plans based on clinical acceptability and provided written feedback on the plan check process. We then created an additional 10 plans, featuring a similar distribution of treatment site and planning errors, which the participants reviewed following a 2‐week break with our customized checklist (Figure 1). After all plan checks had been completed and each plan had been scored, participants were provided with a document summarizing the errors present in each plan and a final, anonymous survey to evaluate the process. The survey collected information about the overall RPA plan quality, the time needed to check each plan, with and without the checklist, the clarity of the RPA plan report, the usefulness of the plan checklist, and any suggestions to improve the checklist or plan review process.

Modifications were made to the checklist to reflect the results of the survey. The checklist was reduced from 90 items to 18 based on feedback that there was substantial overlap with recommendations from AAPM task group 275, leading to redundancy in the plan review process. While the initial checklist contained items for the entire plan check process (RPA output and final treatment parameters), the revised version focused specifically on the review of the RPA output. The majority of the redundant items were removed, excluding basic planning parameters, and all checks related to identifying automatically generated plan failure modes were preserved.

### 2.3 Study 2

**FIGURE 2:**
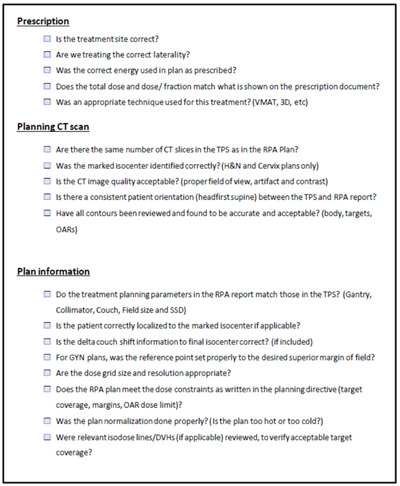
Checklist (version 2). The initial checklist was revised based on feedback from study participants that the checklist had too much overlap with prior clinical practice. The revised version focuses specifically on the errors which could be present during the review of RPA output, as identified in a prior failure mode and effects analysis

A second study was then performed with 14 senior medical physics residents from a variety of CAMPEP‐accredited residency programs within the United States. Participants were again provided with RPA training materials before proceeding with the plan review process. Six of the participants were chosen at random to be provided with the updated checklist (Figure 2) to assist with their review, and eight were given no checklist. This uneven split was caused by participants from the checklist cohort dropping out prior to completing the study and was not intentional. Each resident reviewed 10 automatically generated plans, five of which contained errors. Residents were given 1 month to complete their review to prevent the study from interfering with their clinical training. The final survey was then repeated after all plan reviews had been completed.

## 3 RESULTS

### 3.1 Study 1

**FIGURE 3:**
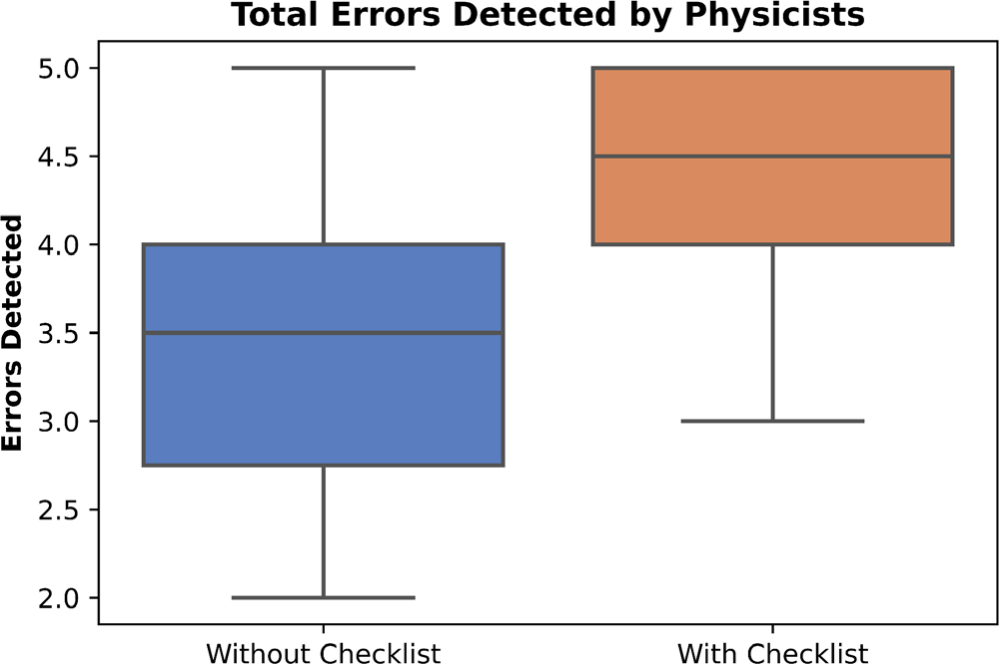
Errors detected without and with the initial checklist in study 1 (physicists)

All eight physicists completed 20 plan checks each, separated into two phases, without and with the customized checklist. Each phase contained five errors to be detected per physicist and 40 errors in total. In phase 1, 27 errors (68%) were detected, and in phase 2, 35 errors (88%) were detected. Without and with the checklist, the mean and standard deviation of errors detected per participant was 3.4 ± 1.1 and 4.4 ± 0.74, respectively (Figure 3). A *t*‐test indicated that the improvement in error detection was statistically significant (*p* = 0.02) for the physicist cohort.

### 3.2 Study 2

**FIGURE 4:**
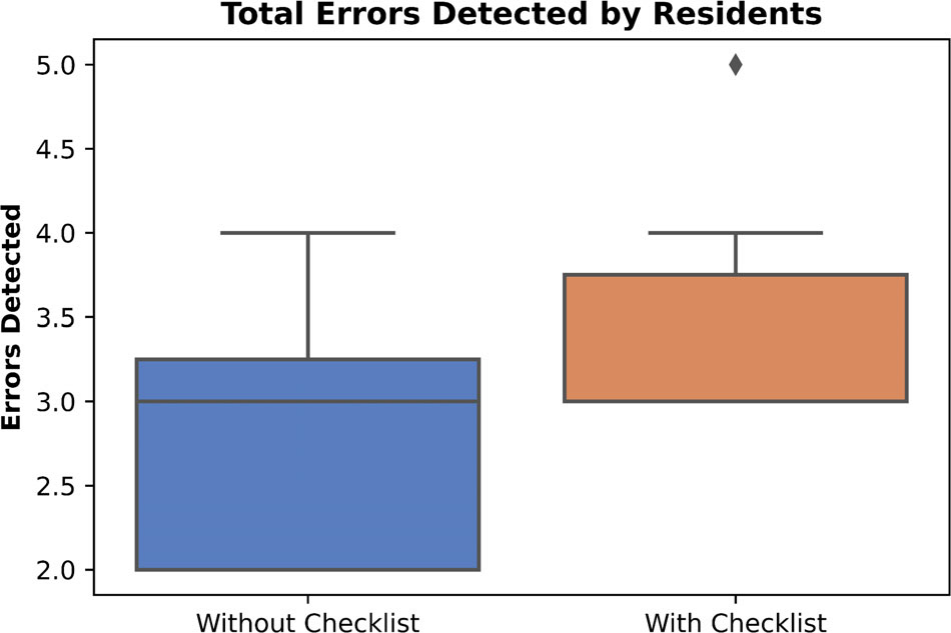
Errors detected without and with the revised checklist in study 2 (residents)

The revised checklist was assessed by 14 physics residents who completed 10 plan checks each, five of which contained errors. Eight residents completed their reviews without the checklist, and the remaining six participants utilized the checklist. Without the checklist, 53% (21 out of 40) of errors were detected; with the checklist, 70% (21 out of 30) of errors were detected. Without and with the checklist, the mean and standard deviation of errors detected per participant was 2.9 ± 0.84 and 3.5 ± 0.84, respectively (Figure 4). A *t*‐test indicated these results were not statistically significant (*p* = 0.08) for the resident cohort, however, the increase in error detection when the checklist was utilized showed that there would be a clinical benefit when using the custom checklist to assist with plan reviews.

## 4 DISCUSSION

We developed a checklist specifically for use when performing physics reviews of automatically generated treatment plans. Two versions of the checklist were developed and tested with different groups of medical physicists and trainees. While the first checklist was found to be effective at increasing the detectability of errors in the treatment plan, the study participants were overwhelmingly dissatisfied with its length. The revised checklist was significantly shorter but still led to a similar improvement in error detection compared to no checklist.

### 4.1 Error detection in physics plan review

Errors in treatment planning, both automated and manual, are inevitable. A study by Gopan et al. found that when physics plan checks were performed on a set of treatment plans containing 113 errors, only 67% of errors were detected at this step of the workflow.^27^ Similarly, Ford et al. found that pretreatment plan review by physicists leads to the detection of 63% of errors.^4^ While these numbers may seem discouraging, Ford et al. also identified that when physics plan review is used in conjunction with other quality assurance checks, such as physician review, portal dosimetry, and therapist review, 97% of errors were detected before impacting the patient. This reinforces that every step of the treatment planning process should be used for quality assurance to increase redundant checks, and limit patient risk.

The rate of error detection for RPA plans when utilizing the custom checklist (88% for physicists and 70% for residents) is higher than in the prior studies, reinforcing that the final treatment plan and plan report from the RPA is clear and errors are evident. We recognize that the rate of error detection would have ideally been higher for both studies (physicists and residents) when utilizing the checklist. However, the improvement in error detection in both cohorts that used the checklist indicates the utility of this quality assurance aid. Physics plan review should never be used as the stand‐alone quality assurance step, and we are confident that when evaluated in conjunction with other stages of the planning process, the rate of error detection will increase.

### 4.2 Participant experience levels

As this checklist will be used by physicists or other clinicians with varying levels of experience, we evaluated its effectiveness in two separate populations of physicists: clinical faculty physicists with more than 2 years of experience in checking radiotherapy plans and therapeutic medical physics residents who were in their second year of a CAMPEP‐accredited residency program.

While the rate of error detection was higher in both participant populations when the checklist was used, we identified a lower rate among the residents in study 2. While this could be a result of a lack of experience or the modifications that were made to the checklist, we also found that on average, the residents reported that they spent less time reviewing each plan than the experienced physicists; therefore, the lower detection rate could be attributed to a less thorough review. Regardless, the rate was higher in both populations with the checklist (20% in the first study and 18% in the second), indicating that it is an impactful quality assurance aid.

### 4.3 Trends in error detection

In study 1, when the custom checklist was not utilized for plan review, we found that physicists were least likely to detect an error in plan normalization, such as a dose that is too high or too low (25% detected). When the custom checklist was utilized, the rate of detection for improper plan normalization increased to 75%.

In study 2, we found that both cohorts (with and without the checklist) were unable to detect when the CTV coverage did not match the intended prescription. Without and with the checklist, 0% and 17% of participants detected this error, respectively. The low detection rate when reviewing CTVs can likely be attributed to the lower clinical experience level of the residents, as CTVs based on nodal regions can be difficult to visually delineate. This same error was detected by 50% and 100% of physicists, without and with the checklist respectively, showing the increase of detection with experience.

Incorrect reference point position and incorrect isocenter detection are two errors that are somewhat unique to the RPA workflow, however, we found that for both studies these errors had the highest detection rates among both the checklist and no checklist cohorts. This highlights a strength of the RPA system—the clarity of the final plan report. When unique errors are easily detectable, this indicates that the presence of the error was effectively displayed on the plan documentation, simplifying the review process.

### 4.4 Survey feedback

In the survey from study 1, we received feedback that the provided checklist was too long from 80% of the physicists. Respondents also indicated that the checklist presented limited utility due to redundancy with recommendations from TG‐275, which inform the standard clinical review process. Thus, the checklist was revised to contain only critical errors that would be more likely to occur with automated planning systems. We expect this checklist to be used as an additional review step for automatically generated plans, in conjunction with the established plan review process Patient information checks in the record and verify system were removed.

Only 60% of participants in the first study reviewed all of the relevant RPA training videos and documentation that they had been provided with. In the second study, only 29% of physics residents reported that they had reviewed all provided training materials. We anticipate that, had all training materials been used, the error detection rate would have increased, and the duration of plan review would have decreased, as the auto‐generated plans would be more easily understood.

Most (83%) participants in both studies indicated that it took less than 30 min to review each plan, both without and with the provided custom checklist. Each participant surveyed reported that overall, the length of plan review was unchanged when using the checklist developed for use with automatically generated plans. We conclude that the use of a quality assurance checklist did not increase the time required to complete the plan review and ultimately increased the rate of error detection; thus, it will be an asset to the physics plan check process.

### 4.5 Checklist development

This checklist was developed to assist with the physics plan review for treatment plans that are generated using automated tools. Rather than reiterating the recommendations that were made in AAPM task group 275, we generated a supporting document that should be used in addition to the standard clinical procedure. The final checklist includes errors that were identified as more commonly occurring in plans generated using artificial intelligence‐based tools and data from a failure modes and effects analysis study.^25^ This decision led to more specific checks and a shorter checklist in the second study.

### 4.6 Future deployment

The final iteration of the custom checklist, included in the appendix, will be deployed to physicists for use with the RPA. Training will be provided to help guide the plan review process, with emphasis on possible high‐risk failures. Users will then be provided with test plans to review using the checklist, several of which will contain previously assessed errors. If all errors are detected, the user will be able to proceed with using the RPA for plan generation. If errors are not detected, additional training will be provided to the user and the checklist will be modified to add any missing items. The final iteration of our checklist will be evaluated as part of an end‐to‐end test of the RPA commissioning and training procedures.

### 4.7 Limitations

This study included a limited number of participants because of the large time commitment required by each volunteer. In the first study, conducted with experienced clinical physicists, each volunteer participated in two rounds of plan checks, first without and then with the customized checklist. This format could introduce observer bias into the results: each participant was familiar with the RPA plan reports and performing plan reviews before the second phase of the study, which could have resulted in a higher number of errors detected with the checklist. To eliminate this factor from the second round of the study, each physics resident was randomly assigned to the checklist or no checklist cohort, and all plan checks were performed in one session.

This study, including the development of a checklist, was created based on the results of a failure modes and effects analysis that focused on the RPA system. Therefore, the checklist will need to be adapted when applied to other systems. Our results confirm that checklists are useful with automated planning approaches, which should apply to other systems, including those that we expect treatment planning system vendors to introduce in future versions.

## 5 CONCLUSION

Our results indicate that the use of a customized checklist in the review of automated treatment plans will result in a higher error detection rate and thus improved patient safety. While this analysis was performed using the RPA as a case study, we anticipate the results will be scalable to other automated systems.

## CONFLICT OF INTEREST

The authors declare no conflict of interest.

## AUTHOR CONTRIBUTIONS

All authors (Kelly A. Nealon, Laurence E. Court, Raphael J. Douglas, Lifei Zhang, and Eun Young Han) assisted with the design, data acquisition, and analysis for this publication. The article draft was written primarily by Kelly A. Nealon and Eun Young Han, however all authors provided feedback and revisions prior to final approval of the article. All authors accept responsibility for the information presented in this article.

## Supporting information

Supporting information
