# Supplementary material for: Development and validation of a checklist for use with automatically generated radiotherapy plans
Source: J Appl Clin Med Phys. 2022 Jun 30;23(9):e13694. doi: 10.1002/acm2.13694 (PMC9512344; doi:10.1002/acm2.13694)
Supplement: Supplementary file 1 — Supporting information [file ACM2-23-e13694-s001.pdf]

Checklist for Review of RPA Output Plans

Prescription / Service Request

- ☐ Service Date
- ☐ Patient Name
- ☐ MRN
- ☐ Patient De-identified ID
- ☐ Treatment Site
- ☐ Disease Extent
- ☐ Laterality
- ☐ Treatment Machine
- ☐ Technique (VMAT, 3D)
- ☐ Energy
- ☐ Total Dose
- ☐ Fractionation
- ☐ Target coverage

CT Scan

- ☐ Marked Isocenter correctly identified (**Head and Neck** or **Cervix** plans)
- ☐ Planning CT date
- ☐ Consistent # of CT slices in RPA & TPS
- ☐ Patient Orientation (HFS)
- ☐ Acceptable CT image quality (artifacts, FOV, etc)
- ☐ Target names
- ☐ Target contours
- ☐ OAR names
- ☐ OAR contours
- ☐ Target margins
- ☐ Body/External Contour

Final RPA Plan

- ☐ Gantry angle
- ☐ Collimator angle
- ☐ Couch angle
- ☐ Planning constraints met
- ☐ Hot Spot Dose
- ☐ Hot Spot in Target
- ☐ RPA QA Checks Passed
- ☐ Shift from marked isocenter to final isocenter verified
- ☐ **If Cervix**: Reference point set to desired superior field margin
- ☐ **If Chest Wall**: Reference point set at desired inferior tangent field margin

RPA and TPS Plans

- ☐ Plan DICOM imported to TPS without error
- ☐ Beam parameters match between TPS and RPA (Gantry angle, collimator angle, table angle, jaw size, etc.)
- ☐ MLC pattern matches between TPS and RPA **(Cervix & Chest Wall)**
- ☐ MU/beam matches between TPS and RPA prior to recalculation.

Recalculate with preset values of MU from RPA

Final Plan Review **MUST be performed in local TPS following plan recalculation**
